# Should we continue searching for the single best PEEP?

**DOI:** 10.1186/s40635-022-00438-7

**Published:** 2022-03-21

**Authors:** Filip Depta, Marko Zdravkovic, Michael A. Gentile

**Affiliations:** 1 Department of Anesthesiology and Intensive Care, East Slovak Institute for Cardiovascular Diseases, Ondavská 8, 040 11 Košice, Slovakia; 2 Faculty of Medicine, Pavol Jozef Šafárik University, Košice, Slovakia; 3 Department of Anesthesiology, Intensive Care and Pain Management, University Medical Centre Maribor, Maribor, Slovenia; 4 Faculty of Medicine, University of Ljubljana, Ljubljana, Slovenia; 5 IPM Chirana Inc, Durham, NC USA

Positive end-expiratory pressure (PEEP) has been an important component of mechanical ventilation for over 50 years, yet PEEP selection remains highly debatable in the critical care community. Despite numerous studies trying to answer the ‘PEEP question’, there are often conflicting opinions when it comes to recommendations based on clinical trials [1].

Three major studies (ALVEOLI, LOVS, EXPRESS) assessing higher versus lower PEEP in combination with low tidal volume (Vt) for acute respiratory distress syndrome (ARDS) failed to show decrease in mortality, although a meta-analysis of the three trials suggested a survival benefit for a subgroup of patients with severe ARDS [2]. It is universally accepted that higher PEEP may be helpful in ARDS patients where recruitability is expected but proves harmful in other groups of patients contributing to ventilation induced lung injury (VILI). Therefore, as many authors have suggested that not only one PEEP does not fit all, but also looking for the ‘best’ PEEP has little meaning because within mechanical power, it is just one part of the whole [3].

Since the introduction of PEEP into clinical practice, researchers have been focusing on finding the single most suitable PEEP that usually corresponded to best lung mechanics (i.e., highest compliance of the respiratory system) or oxygenation. However, we would like to challenge this idea and introduce another approach to PEEP research in critically ill lungs. We hypothesize, that patients with nonhomogeneous lungs could be supported on mechanical ventilation using multiple and alternating levels of PEEP. In the following paragraphs, we will list and elaborate on several reasons supporting our hypothesis of potential benefits of using multiple alternating PEEP levels.

First, Suter demonstrated similar respiratory system compliance (C_RS_) at different PEEP levels that depended on the Vt delivered [4]. Data from our research on COVID-19-related ARDS identified rather a range of suitable PEEP levels where most parameters of lung mechanics during pressure-controlled ventilation show very similar values (Fig. 1). This range of PEEP levels was relatively wide: 5–8 cm H_2_O. Range of suitable PEEP levels was also reported by other authors assessing driving pressure using volume-controlled ventilation with different Vt (4, 6 and 8 ml/kg of predicted body weight [PBW]) [5].

**Fig. 1:**
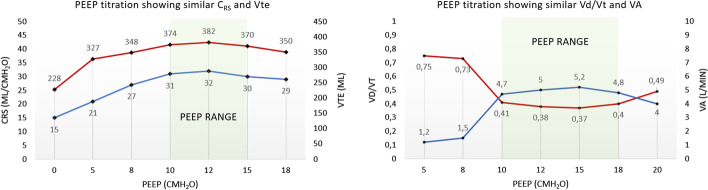
PEEP titration using constant inspiratory pressure of 14 cmH_2_O during pressure-controlled ventilation (PCV) in two different passive severe COVID-19 ARDS patients. Colored boxes are pointing out similar lung mechanics at different PEEP levels. Vte—expiratory tidal volume, C_RS_—respiratory system compliance, Vd/Vt—dead space fraction, VA—alveolar ventilation, PEEP—positive end-expiratory pressure

Second, the degree of recruitability varies widely in patients with ARDS. If constant inspiratory pressure is applied during the ascending PEEP levels and increase in Vt is observed, lungs are considered recruitable [6]. Given the previous studies and current knowledge, what PEEP level should be chosen if the lungs show evidence of similar recruitability at more than just one PEEP level? Furthermore, what deviation from the ‘best’ value is considered significant to guide the most appropriate PEEP selection? This is the range of suitable PEEP levels that show very similar values in the supposedly recruitable zone. Should the highest PEEP where best compliance is observed be selected, or rather the lowest PEEP with still satisfactory oxygenation?

Third, ventilation with single PEEP level within recruitable zone of ARDS lungs means that some alveoli will not be recruited, and some will be overdistended with respect to pathophysiology of the ‘baby lungs’, including different regional time constants and opening airway pressures [7].

Fourth, alveolar dynamics continues to represent a “black box” of mechanical ventilation. Intra-tidal collapse has been rightfully feared to contribute to VILI. However, it has been shown that intra-tidal collapse and decollapse was similar at PEEPs of 5 and 15 cmH_2_O in ARDS patients and that PEEP as high as 15 cmH_2_O did not prevent cyclic alveolar opening and closing [8]. Alveolar collapse happening during tidal ventilation with presumed sufficiently high PEEP is likely because the single best PEEP only represents the best tradeoff between recruitment and overdistension.

As Gattinoni et al. stated, “The best PEEP does not exist. To pretend and claim that we may find a PEEP level that avoids intra-tidal recruitment–derecruitment, providing in the meantime the best compliance, best oxygenation and lowest dead space, without causing hyperinflation and affecting hemodynamics, reflects a wishful dream that has nothing to do with the reality” [9].

Perhaps alternating multiple levels of PEEP should be the parallel focus of research where similar lung mechanics are identified. Reasons to use alternating multiple levels of PEEP during mechanical ventilation while delivering controlled or supported breaths at each PEEP level, are:

1. It was shown on a 5-compartmental model of nonhomogenous lung that the part of Vt is redistributed from shorter to longer time constant compartments when using multiple levels of PEEP [10]. In clinical practice, this may represent better aeration and decrease in ventilation inhomogeneities.
2. Higher PEEP is not sustained and therefore negative consequences (i.e., higher mechanical power delivered to the lung tissue or negative impact on hemodynamics) might not be so pronounced.
3. If the recruitable zone of the ARDS lungs is larger than delivered protective Vt (i.e., 6 ml/kg/PBW), then using constantly alternating multiple PEEP levels might allow improved recruitment without significantly increasing driving pressure (i.e., protective Vt is delivered at different PEEP levels, thus ventilating bigger part of recruitable zone of ARDS lung).

Therefore, if we acknowledge that intra-tidal collapse cannot be avoided and also know that ‘highest’ compliance, ‘best’ oxygenation and ‘lowest’ Vd/Vt, all occur at different PEEP levels, should research focus continue to seek the single best PEEP or rather consider a different approach? Targeting recruitable zone of the nonhomogenous lungs using multiple alternating PEEP levels throughout the acute phase of ARDS, might propose an alternative approach on which to focus further research efforts.

## Data Availability

Any data-related questions should be directed to the corresponding author.

## Abbreviations

ARDS: Acute respiratory distress syndrome
COVID-19: Coronavirus disease 2019
C_RS_: Respiratory system compliance
PBW: Predicted body weight
PCV: Pressure-controlled ventilation
PEEP: Positive end-expiratory pressure
VA: Alveolar ventilation
Vd/Vt: Dead space fraction
VILI: Ventilator-induced lung injury
Vt: Tidal volume
Vte: Expiratory tidal volume
